# Invasion and Dispersal of *Biomphalaria* Species: Increased Vigilance Needed to Prevent the Introduction and Spread of Schistosomiasis

**DOI:** 10.3389/fmed.2021.614797

**Published:** 2021-02-10

**Authors:** Mohamed R. Habib, Shan Lv, David Rollinson, Xiao-Nong Zhou

**Affiliations:** ^1^Medical Malacology Laboratory, Theodor Bilharz Research Institute, Giza, Egypt; ^2^National Institute of Parasitic Diseases, Chinese Center for Diseases Control and Prevention, Shanghai, China; ^3^Key Laboratory of Parasite and Vector Biology, Ministry of Health, Shanghai, China; ^4^National Center for International Research on Tropical Diseases, Shanghai, China; ^5^WHO Collaborating Center on Tropical Diseases, Shanghai, China; ^6^School of Global Health, Chinese Center for Tropical Diseases Research, Shanghai Jiao Tong University School of Medicine, Shanghai, China; ^7^Department of Life Sciences, Natural History Museum, London, United Kingdom

**Keywords:** *Biomphalaria*, invasion, *Schistosoma mansoni*, schistosomiasis, transmission

## Abstract

Biological invasion is a matter of great concern from both public health and biodiversity perspectives. Some invasive snail species may trigger disease emergence by acting as intermediate hosts. The geographic distribution of *Schistosoma mansoni* depends on the presence of susceptible species of *Biomphalaria* freshwater snails that support the parasite's transformation into infective stages. *Biomphalaria* spp. have shown strong local and global dispersal capacities that may increase due to the global warming phenomenon and increases in the development of agricultural and water projects. Should intermediate hosts become established in new areas then this will create potential transmission foci. Examples of snail invasions that have had an impact on schistosomiasis transmission include the introduction of *Biomphalaria tenagophila* to Congo and *B. glabrata* to Egypt. The current spread of *B. straminea* in China is causing concern and needs to be monitored closely. An understanding of the mode of invasion and distribution of these snails as well as their experimental susceptibility to *S. mansoni* will predict the potential spread of schistosomiasis. Here we review the invasion patterns of *Biomphalaria* snails and factors that control their distribution and the impact that invasion may have on intestinal schistosomiasis transmission. In addition, we propose some possible surveillance responses for optimum control strategies and interventions. Whenever possible, swift action should be taken to contain any new occurrence of these intermediate snail hosts.

## Background

Aquatic invasion by exogenous species can cause native biodiversity loss and deleterious environmental and economic impacts (1). Biological invasions of gastropod molluscs have been strongly linked to serious problems to human health. They can cause expansion, emergence, or re-emergence of infectious diseases (2). These risks are anticipated to intensify due to ongoing climate change and global warming which continue to create new suitable freshwater environments for snail species serving as disease carriers (3). This review will consider the invasion patterns of *Biomphalaria* spp. (Class: Gastropoda; Family: Planorbidae) that act as the intermediate hosts for *Schistosoma mansoni*, a trematode parasite causing intestinal schistosomiasis. *Biomphalaria* are widely distributed in sub-Saharan Africa, South America, and other subtropical regions. Several species of *Biomphalaria* have shown strong local or global dispersal capacities. Numerous accidental or deliberate introductions of *Biomphalaria* spp. have been reported in tropical areas (4). *Biomphalaria* dispersal is of prime interest because it is often associated with the creation of new transmission foci for schistosomiasis. *B. glabrata, B. straminea, B. tenagophila*, and *B. pfeifferi* are the most common invasive species that have been introduced into new areas outside their native habitats and acted as potential intermediate hosts for schistosomiasis transmission (5). Therefore, an extensive knowledge of the geographic distribution of susceptible species of *Biomphalaria* is of considerable importance for the control of schistosomiasis and its epidemiologic surveillance as well as for future delineation of potential risk areas.

## Geographic Distribution of Susceptible Species of *Biomphalaria*

The global distribution map of *S. mansoni* is largely defined by the occurrence of *Biomphalaria* species that are able to transmit the parasite. According to DeJong et al. (6), there are 34 species of *Biomphalaria* distributed worldwide. Looking back at the long evolutionary history of *Biomphalaria* spp. it is clear that invasions into new geographical areas have been a regular feature. Molecular evidence suggests that a *B. glabrata* like taxon, South American in origin, dispersed across the Atlantic to Africa and gave rise to the African species possible quite recently in the Plio-Pleistocene (1.8–3.6 Myr ago). This West to East transoceanic dispersal could have occurred in the feathers of aquatic birds or on vegetation rafted across the ocean followed by successful colonization (7).

Four Neotropical species act as intermediate host for *S. mansoni* in the wild, these includes, *B. glabrata* in Antigua, Brazil, Curacao, Dominica, Guadeloupe, French Guiana, Haiti, Saint Kitts and Nevis, Martinique, Montserrat, Puerto Rico, Dominican Republic, Saint Lucia, Suriname, and Venezuela, *B. tenagophila* in Southern Brazil, Argentine, Paraguay, Uruguay, Peru, and Bolivia, *B. straminea* in North-Eastern Brazil, Venezuela, Suriname, French Guiana, Guyana, Peru, Paraguay, Argentine, Dominica, Grenada, Guadeloupe, Martinique, Dominican Republic, Trinidad, Uruguay, and Costa Rica and *B. prona* in Venezuela (8, 9). Other species showed susceptibility to experimental infection, but have not been found naturally infected, such as *B*. *amazonica* found in Brazil, Bolivia, and Colombia (10), *B. havanensis* found in Haiti, Mexico, Puerto Rico, Cuba, and Venezuela, *B. helophila* found in Peru, Cuba, Costa Rica, Guatemala, Belize, Haiti, Mexico, Saint Thomas, El Salvador, Dominican Republic, Puerto Rico, Barbados, and Nicaragua (11), *B. peregrina* from Ecuador, Bolivia, Chile, Brazil, Paraguay, Peru, Uruguay, Argentine, and Colombia (12), and *B. sericea* from Ecuador (13). Both susceptible and refractory species may coexist in the same municipality. In São Paulo, in the Southeast Region of Brazil, *B. occidentalis, B. oligoza, B. peregrina, B. schrammi, B. straminea* and *B. tenagophila* are present in water bodies of the upper basin of the Tietê River (14).

**Figure 1:**
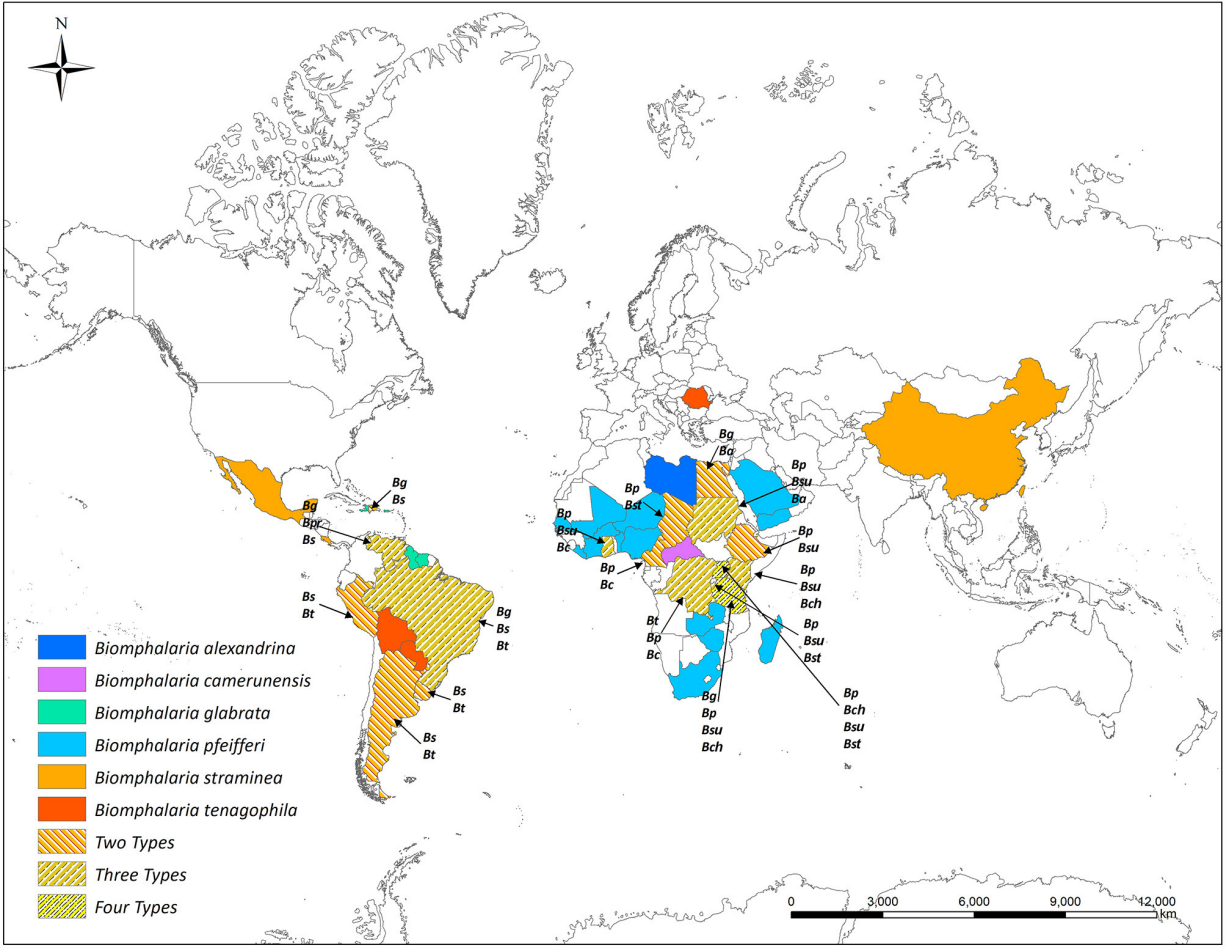
Global distribution of susceptible species of *Biomphalaria* (country level). *Ba, Biomphalaria alexandrina*; *Bc, Biomphalaria camerunensis*; *Bch, Biomphalaria choanomphala Bg, Biomphalaria glabrata*; *Bp, Biomphalaria pfeifferi*; *Bpr, Biomphalaria prona*; *Bs, Biomphalaria straminea*; *Bst, Biomphalaria stanleyi*; *Bsu, Biomphalaria sudanica*; *Bt, Biomphalaria tenagophila*.

Most African species of *Biomphalaria* are known as active intermediate hosts of schistosomes in the field (15). Numerous records of natural infections of *Biomphalaria* with *S. mansoni* in many African countries are present in literature, examples include *B. alexandrina* in Egypt (16, 17), *B. camerunensis* in Cameroon (18), *B. choanomphala* in Tanzania, Kenya, and Uganda (19–21), *B. sudanica* in Tanzania and Uganda (19, 22), Kenya (23), Ethiopia (24), Burundi (25), and Sudan (26), *B. stanleyi* in Uganda and Tanzania (19, 22), and *B. pfeifferi*, the most important intermediate host for *S. mansoni* in Africa due to its ubiquitous distribution, was found infected in Burundi (25), Cameroon (27), Mali (28), Niger (29), Senegal (30), South Africa (31), Sudan (32), Zimbabwe (33), Tanzania, Uganda (19), and Benin (34). In addition to the geographic areas mentioned above for naturally infected snails, *Biomphalaria* spp. are widely distributed in many other wetland areas throughout the Americas and Africa (Figure 1 and Table 1).

**Table 1:** Distribution of naturally susceptible species of *Biomphalaria*.

**Species**	**Geographic distribution (Country level)**	**References**
*Biomphalaria glabrata*	Brazil*	(8, 35)
	French West Indies	(36, 37)
	West Indian Island of St. Lucia	(38)
	Venezuela	(39, 40)
	Lesser Antilles	(40)
	Puerto Rico	(41)
	Haiti	(42)
	Dominican Republic	(9, 43)
	Surinam	(9)
	French Guiana	(9)
	Egypt	(44–46)
*Biomphalaria straminea*	Brazil	(47–49)
	Hong Kong	(50, 51)
	China	(52–54)
	French West Indies	(36)
	Uruguay	(55, 56)
	Venezuela*	(9, 57)
	Costa Rica	(58)
	Argentina	(59, 60)
	Dominican Republic	(61, 62)
	Mexico	(63)
	Peru	(64)
	Dominica	(65)
*Biomphalaria tenagophila*	Romania	(66)
	Brazil	(67–69)
	Uruguay	(70, 71)
	Paraguay	(72)
	Argentina*	(73, 74)
	Peru	(75)
	Democratic Republic of Congo	(9, 76)
	Bolivia	(9)
*Biomphalaria pfeifferi*	South Africa*	(15)
	Ethiopia	(77–79)
	Nigeria	(80, 81)
	Sudan	(82, 83)
	Saudi Arabia	(84)
	Ghana	(85)
	Democratic Republic of Congo	(86, 87)
	Senegal	(88, 89)
	Kenya	(90, 91)
	Zimbabwe	(92)
	Niger	(93)
	Mali	(94, 95)
	Liberia	(96, 97)
	Cote-d'Ivoire	(98)
	Tanzania	(99)
	Benin	(100)
	Cameroon	(101, 102)
	Burundi	(25)
	Yemen	(103)
	Zambia	(104, 105)
	Chad	(106)
	Uganda	(107)
	Madagascar	(15)
	Burkina Faso	(108)
*Biomphalaria alexandrina*	Egypt*	(109–111)
	Libya	(112)
	Sudan	(113)
*Biomphalaria sudanica*	Sudan*	(15)
	Tanzania	(114, 115)
	Kenya	(91)
	Uganda	(19, 116)
	Ghana	(117)
	Ethiopia	(118)
	Burundi	(25)
*Biomphalaria choanomphala*	Tanzania*	(15)
	Uganda	(119)
	Kenya	(120)
*Biomphalaria camerunensis*	Cameroon*	(121)
	Democratic Republic of Congo	(122)
	Ghana	(15)
	Central African Republic	(15)
	Zaire	(123)
*Biomphalaria prona*	Venezuela*	(9, 124)
*Biomphalaria stanleyi*	Tanzania*	(19, 114)
	Uganda	(22)
	Burundi	(125)
	Chad	(126)

* *Indicates first record of the species*.

## Ecological Factors Controlling *Biomphalaria* Abundance and Distribution

Many ecological or environmental factors regulate the distribution and abundance of *Biomphalaria* spp. The high fecundity of *Biomphalaria* and their ability to self-reproduce are important traits that support their successful invasion. Adult snails can produce 10,000 eggs per year. The egg-to-egg period requires approximately 11 weeks (127). With a life span of up to one year and a half, *Biomphalaria* can undergo several generations over a year (128). Following embryonic hatching from egg capsules, *Biomphalaria* undergo a series of developmental steps leading to maturation and reproduction. The snail has a great capacity to live and reproduce under different environmental conditions (129, 130). *B. glabrata* and *B. straminea*, for example, were found in 10 of 11 different types of aquatic habitats in Brazil (131).

In natural habitats, *Biomphalaria* are under the influence of several ecological factors that control the population density of snails although temperature is a key determinant. Detailed review of environmental factors conditioning the habitat of *Biomphalaria* can be found in Malek (132), Brown (15), and Rollinson (133). In summary, these ecological factors can be divided into biotic and abiotic factors.

### Biotic Factors

Biotic factors such as plants and food supply have a particular influence on the local abundance and distribution of snails. Predator and competitor species such as other species of snails, fish and insects also control snail populations. Data from field studies in Africa and Brazil indicate that Tilapia fish and some Dipteran insect's larva are predators of *Biomphalaria* in fishponds and lakes (134). In some cases, the absence of these natural predators may have a detrimental impact on schistosomiasis transmission. For example, the construction Diama Dam on Senegal River Basin obstructed the annual migration of native river prawns, *Macrobrachium vollenhoveni*, that feed on schistosomes intermediate host snails, leading to massive outbreak of schistosomiasis. Indeed, field experiments showed that reintroduction of the prawns caused a significant reduction in infected snails and human schistosomiasis prevalence (135, 136). Moreover, several pilot control studies involving competitor snails, such as *Pomacea glauca, Marisa cornuarietis, Melanoides tuberculata*, and *Helisoma duryi*, achieved varying results; characterized mostly by ecological upsets after initial snail reductions (137, 138).

### Abiotic Factors

Abiotic factors, such as temperature and water chemistry, can have evident effects on *Biomphalaria* spp. over short distances within a single waterbody (15). In particular, temperature is important for snail survival and reproduction. Variation in water temperature affects distribution of snails not only from season to season but also from year to year (139). Recent studies on the effects of temperature changes on the growth, fecundity and survival of intermediate host snails of schistosomiasis and schistosomes prevalence indicated that temperature increase may alter the distribution, breeding, growth and survival of snails, and consequently may increase the spread of schistosomiasis (140–142). In Egypt, high temperatures appear to reduce snail breeding and March is the month of maximal breeding for *B. alexandrina* (143). Yousif et al. (144) found that *B. alexandrina* populations exhibited two peaks of populations density that differ in heights depending on the extent of water temperature and winter closing. The first peak is from April-May and the second is from November-December. These two peaks are permeated by two lows following the Winter Closure and during the hot summer season. Under laboratory condition, *B. alexandrina* has optimal growth and egg laying at 26–28°C (145). A similar temperature range was also found with *B. glabrata* (146). Also, a positive correlation was observed between *B. sudanica* abundance and water temperature (22). Appleton et al. (147) observed a negative correlation between fecundity and increasing above-optimal temperatures (>27°C) during maturation period of *B. pfeifferi*. The authors concluded that the change in temperature regime is responsible for the species' absence from pans in some areas of South-Eastern Africa due to the sensitivity of *B. pfeifferi* to high temperature. Furthermore, the distribution of *B. pfeifferi* and *B. sudanica* is limited to north and north-eastern parts of Uganda characterized by suitable temperatures (148). Temperatures higher than optimal values cause retardation in the development of gonads and albumen gland and a decrease in egg-production and viability.

Water chemistry is an important factor for snail distribution (149). For example, inorganic salts govern the osmotic properties of the aquatic environment, which in turn are linked to the sensitivity of snail eggs to salinity. Most common ions in natural water include calcium, magnesium, sodium, bicarbonate, carbonate, chlorine, and phosphate. Snails usually prefer environments rich with calcium. The presence of calcium in water is important for snails since it is essential for the construction of the shells (150). Studies on *B. pfeifferi* showed that the snail is restricted to water with a minimum calcium concentration of 5 mg/l. High ratios of magnesium/calcium and sodium/calcium indicate low bioavailability of calcium and exert a negative impact on the egg production of the snails and can reduce their densities (151). Calcium has been associated with high fecundity of *B. glabrata* (149). El-Khayat et al. (152) showed that the habitat preferred by *B. alexandrina* snails contained higher concentration of various common ions such as potassium, sodium, and calcium. Also, field studies by Kloos et al. (35) showed that the widespread distribution of *B. glabrata*, and its infection with *S. mansoni*, together with differences in the seasonal distribution of snails is correlated to high calcium levels that appear to promote large *B. glabrata* populations in wells and springs.

Other environmental conditions such as turbidity and water pollution have an influence on snail abundance. Becker et al. (153) found that turbidity and pesticide pollution, at concentrations similar to or higher than those considered safe in environmental risk assessment, are significant factors in increasing the incidence of *B. pfeifferi* and *Bulinus africanus*, the intermediate host for *Schistosoma haematobium*, the parasite responsible for urogenital schistosomiasis, and hence the likelihood of schistosomiasis transmission. Declines in snail competitors and predators, that are less tolerant to pesticides, increase the availability of food for intermediate snail hosts and decrease their mortality rate (154).

## Invasion Patterns

### Long Distance (Global) Dispersal

**Figure 2:**
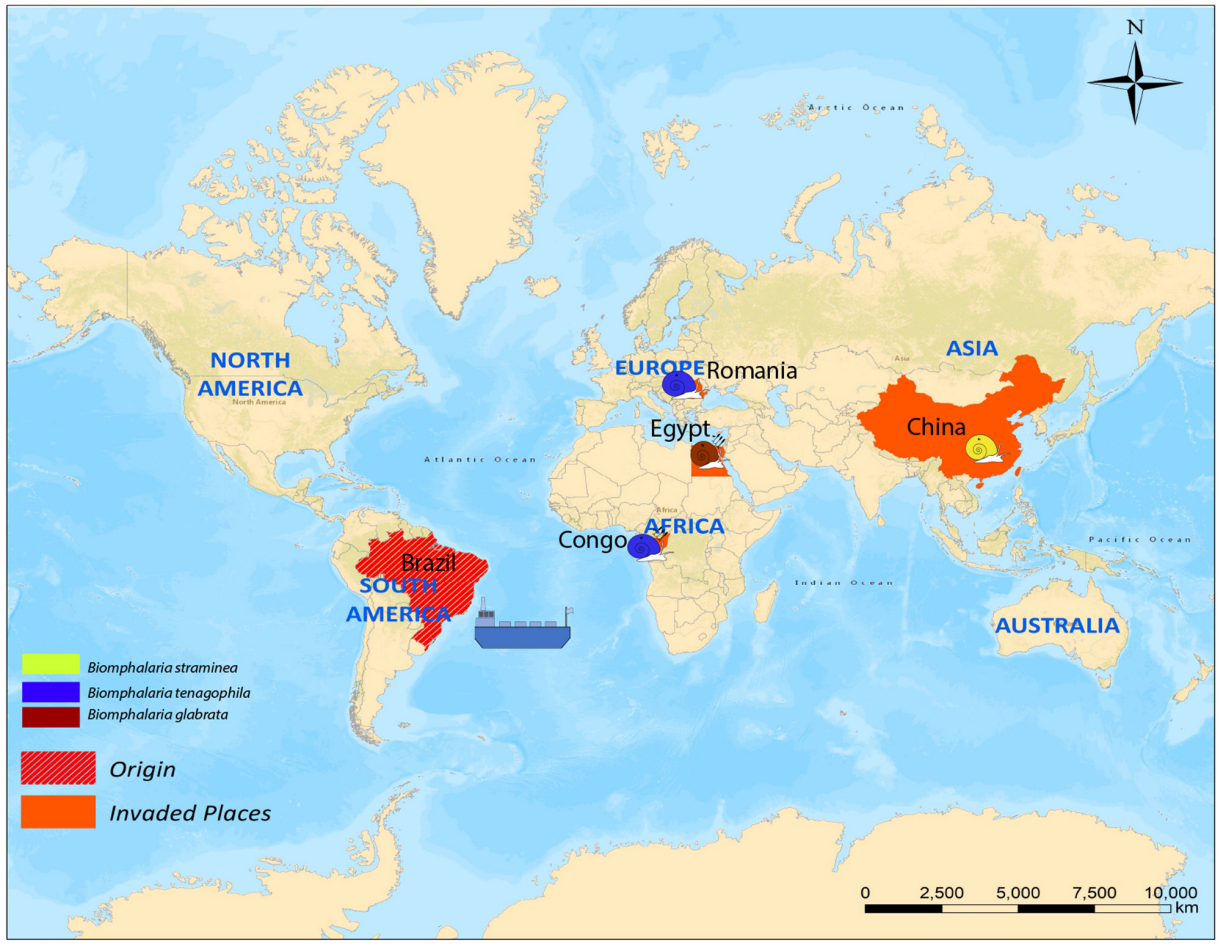
Cases of long-distance dispersal of different *Biomphalaria* species.

There are four cases of long-distance dispersal present in the literature; one case was reported for *B. straminea* (Caribbean islands, China), one for *B. glabrata* (Egypt), and two cases for *B. tenagophila* (Romania and Congo). The dispersal of both *B. glabrata* and *B. tenagophila* (Congo) was implicated in local schistosomiasis transmission (Figure 2).

#### Biomphalaria straminea

*B. straminea*, one of the intermediate hosts for *S. mansoni* in South America, expanded its geographic range to colonize almost all the Caribbean islands (155). In Asia, *B. straminea* was first identified in Hong Kong water systems in the south of China in 1974 (50), in the subsequent years, the snails showed a dispersal capacity in different water courses in southern China. The snail species was found in numerous water bodies in Shenzhen city of Guangdong province, China in 1981 (156), and in the next 2 years showed a growing distribution in Shenzhen rivers, suggesting a spread from Hong Kong rivers systems (52).

Morphological and molecular study for seven populations of *Biomphalaria* snails collected in Guangdong indicated that five populations were closely related to *B. kuhniana*, another South American species refractory to schistosomes infection, the other two populations were identified as *B. straminea* and were more relative to *B. straminea* from Brazil (157). Controversially, further anatomical and molecular investigation of six *Biomphalaria* populations from Guangdong showed a close affinity between these populations and *B. straminea* from Brazil (158). Supporting the latter finding, a more recent study of *B. straminea* in Hong Kong identified two morphotypes of the species; black- and red-colored shell morphs of *B. straminea* in different districts in the new territories in Hong Kong, including places close to the mainland China border. Morphological and molecular analyses of the Hong Kong *B. straminea* showed that they are genetically indistinguishable and are similar to those obtained in mainland China and South America (159). The presence of *B. straminea* in Guangdong may be the result of multiple and different introduction routes and/or peripheral dispersal from Hong Kong populations via passive transport or container ship traffic or naturally due to connections between the water systems of the two adjacent localities (160).

**Figure 3:**
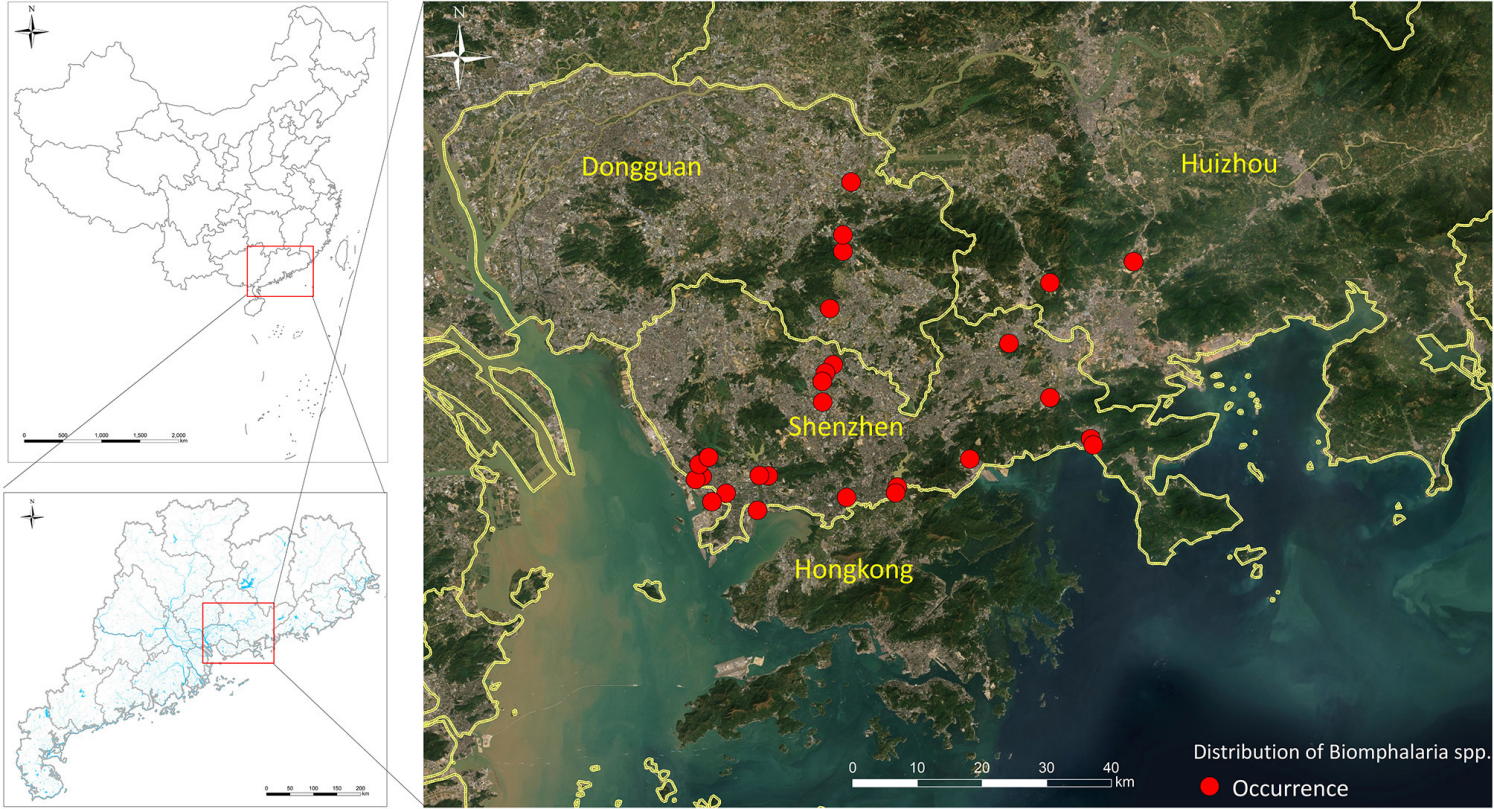
*Biomphalaria straminea* distribution in the south part of China.

The spread of *B. straminea* from Hong Kong to Guangdong confirms its ability to survive and form new colonies in mainland China and its capacity to spread along the rivers (161). *B. straminea* can adapt to drought periods and higher temperatures, an adaptability that will promote its spread to wider geographic areas in China in view of global warming and continued rise in the mean annual temperature of China (162). Further evidence is the recent invasion of the snail to Guanlan and Dasha rivers in Shenzhen city, Guangdong province, China (53). Moreover, recent maps for realized (Figure 3) and predicted spatial distributions of *B. straminea* in southern China, based on Geographical Information Systems (GIS) and species distribution modeling software (MaxEnt), showed the presence of *B. straminea* in many water habitats in Guangdong province and indicated possible suitable habitats in its surrounding provinces such as Guangxi and Fujian (163).

#### Biomphalaria glabrata

*B. glabrata* was maintained in a laboratory setting in Egypt to conduct *Schistosoma* research from where it then allegedly escaped and was recorded from the field in the neighborhood of Cairo in 1981. Later on, in 1996 the snails were collected from water courses in Giza, Qalyoubiya and Kafr El-Sheikh governorates. Being an intermediate host of *S. mansoni*, its escape was considered a serious threat to public health in Egypt (44). In the following years, malacological surveys showed the invasion of the Nile Valley with hybrid snails resulted from mating of *B. alexandrina* and *B. glabrata*, distributed in El-Menia, El-Dakahliya, and Fayoum governorates. The hybrid snails were found naturally infected with *S. mansoni* (164). However, molecular investigations of *Biomphalaria* species collected from areas toward the north of Egypt, the Nile Delta and south of Egypt during 2002-2009 using species specific primers for *B. alexandrina* and *B. glabrata* did not identify *B. glabrata* in the collected populations (165, 166). Recent mapping of *S. mansoni* prevalence in the Nile Delta region showed high infection rates in schoolchildren (167) suggesting a recent contact with areas manifested with infected *Biomphalaria*. This merits further molecular analysis for Egyptian populations of *Biomphalaria* using mitochondrial and genomic sequences to identify species involved in *S. mansoni* transmission and to understand their role and biology of infection.

#### Biomphalaria tenagophila

The invasion of Neotropical snail *B. tenagophila* to Africa was reported from Kinshasa, Democratic Republic of Congo as confirmed by conchological, anatomical, and molecular studies. It was proposed that this species was introduced at the end of the 1960s (5). In Europe, *B. tenagophila* snails had been found in 2004 at Răbăgani, Romania, Eastern Europe but had been misidentified as the common European species, *Planorbarius corneus* (168). In the period between 2005 and 2007, the snail was collected from the same location and its identity was confirmed via morphological and molecular characterization that proved it as *B. tenagophila*. Molecular data obtained from partial mitochondrial 16S ribosomal RNA gene amplification showed 99.74% similarity to *B. tenagophila* originated from Brazil. *B. tenagophila* is not only introduced, but also established in Răbăgani area in Romania. The snail's route of introduction is unknown but it is more likely to be introduced through migrating birds or by plants used in aquariums (66).

### Peripheral Dispersal

Continued modifications of humans to aquatic environments through constructions of major reservoirs and hydroelectric dams and expanding irrigation schemes, quarries, drainage ditches, and aquaculture ponds, improve and create new freshwater bodies and potential snail habitats. *Biomphalaria* spp. rapidly colonize and establish dense populations in such habitats, which in turn facilitate schistosome transmission leading to disease outbreak (136, 169–171). Snails can be passively dispersed by floating aquatic plants or by human activities over the same lakes and rivers. For example, the invasive floating plant *Salvinia* was responsible for *B. pfeifferi* colonization in Lake Kariba in Zambia (172). Passive dispersal of *Biomphalaria* can also be delivered by water currents both during flooding, where water velocity is high so it sweeps away adhering snails, and also by slower drifting because of the habit of most pulmonates to detach and rise to the surface looking for food or as a deliberately dispersive behavior. In an experiment by Dazo et al. (143) they found that *Biomphalaria* and *Bulinus* were recovered as far as 4 km downstream after 1 day following release into a vegetation-free canal flowing rapidly at 0.42 ms^−1^. Field observations from some localities in Brazil indicate that seasonal distribution of *B. glabrata* is characterized by generally lower snail densities in stagnant waters during the dry season however, higher densities in streams and canals during that time appear to be determined largely by the reduction of stagnancy through the addition of rainwater in the former and the flushing out of snails by seasonally increased water velocity in the latter (35). Many records of peripheral dispersal of different *Biomphalaria* spp. in many countries are found in the literature.

## Consequences of Invasions

### Supporting Transmission of *S. mansoni*

The problem with invasion of *Biomphalaria* susceptible to schistosomiasis is that one snail can undergo self-fertilization and act as a founder of an entire colony. Because an individual snail carries only part of the total genetic variation of its original population, the colonies established by the recent dispersal of that snail or even a small group of snails are expected to show reduced genetic diversity compared with their parental population (173). For example, *B. alexandrina* lost allelic diversity at several polymorphic loci after colonizing the Upper Nile in Egypt coming from the Nile Delta region (173). Increased genetic diversity of *Biomphalaria* tends to reduce the overall parasite transmission rate, supposedly due to a dilution effect where in the presence of high genetic diversity it is more likely that some snails are naturally resistant to *Schistosoma* infection, whereas reduced host genetic diversity could benefit *S. mansoni* through an overall increase in transmission rate (174).

Following the introduction of *B. tenagophila* to Kinshasa, Congo in 1970, *S. mansoni* infected specimens were collected from the same area indicating the involvement of the snail in *S. mansoni* transmission and not a native African *Biomphalaria* (76, 175). This finding was confirmed based on a previous experience from Brazil where the first transmission site of *S. mansoni* through using *B. tenagophila* as intermediate host was discovered in the Paraiba valley, state of São Paulo. In the subsequent years, schistosomes showed a great adaptation to the new intermediate host and further spread throughout the valley (8).

*B. pfeifferi* has also been implicated in the increased prevalence of intestinal schistosomiasis in many countries following an extension of its distribution as exemplified by the rise of the disease in Malagasy high plateau, Madagascar after its colonization by *B. pfeifferi* (176, 177). In Egypt, *B. glabrata* was reported in long distances of water courses in Qalyoubiya, Giza, and Kafr El-Sheikh governorates (44, 46). Although no infected snails were collected, laboratory studies on the first generation obtained from field-collected specimens showed its susceptibility to a local strain of *S. mansoni* originally from Giza. The infection rate of *B. glabrata* snails was lower than that of *B. alexandrina*. Also, the incubation period was considerably shorter in *B. alexandrina* than *B. glabrata* which reflects the higher compatibility of the indigenous snails to local parasites. However, total number of cercariae produced per snail throughout the shedding period was higher in *B. glabrata* than in *B. alexandrina*. The results of experimental infection indicated that *B. glabrata* is as efficient as *B. alexandrina* in transmitting local *S. mansoni* parasites (46). The situation of *B. glabrata* became more complicated because the snail was able to hybridize with the local *B. alexandrina* snails producing a susceptible hybrid lineage. Some of the hybrid snails collected from El-Gharbia, El-Behierah, El-Menia, Kafr El-Sheikh, Dakahlia, and Alexandria governorates were found naturally infected with *S. mansoni* (45). Field and laboratory studies suggested that *B. glabrata* and hybrid snails have a higher potential in transmitting *S. mansoni*, which could lead to an increase in schistosomiasis in associated communities (178).

The invasion of Upper Egypt with *B. alexandrina* following the construction of the High Dam has led to outbreaks of *S. mansoni*, with tremendous local increase in the prevalence levels in just a few years (179). Also, as a consequence of *B. alexandrina* introduction to newly reclaimed areas, epidemiological surveys on resident and settler populations showed 49.3% and 40% prevalence of *S. mansoni* in El-Manayef and El-Morra areas, respectively in Ismailia governorate. The seasonality of *Biomphalaria* infection in the new areas showed the presence of two peaks in August and November. These results confirmed the occurrence of transmission of *S. mansoni* which indicated that reclamation of parts of the desert using the Nile water had led to spread of schistosomiasis to these areas (45).

In Northeastern Brazil, a competitive displacement of *B. glabrata* by *B. straminea* occurred after the introduction of the latter species. This displacement was considered as a biological control measure since the introduced snails were thought to be resistant to *S. mansoni* infection and have a greater fecundity (180). However, field data from some endemic areas showed that human infection increased from 35.5 to 61.9% in the locality occupied by *B. straminea*, and decreased from 40.3 to 20.8% in that occupied by *B. glabrata*. These data suggest that *B. straminea* is involved in the transmission of schistosomiasis (181).

Transmission of schistosomiasis can't occur in the absence of a competent intermediate host snail, but once the obligate host has established, schistosomiasis transmission becomes a possibility (2). Although no schistosome larvae have been detected in *B. straminea* or *B. tenagophila* introduced to China and Europe (Romania), respectively, there is still a risk for intestinal schistosomiasis to be locally transmitted into these new geographic zones. The two species are susceptible intermediate host for *S. mansoni* and have been implicated in intestinal schistosomiasis transmission in their native habitats. Thus, global climate change and the possibility of the introduced snails to be in contact with schistosomes, could pose a public health risk. Human travel between countries for business, education or tourism made it possible to find schistosomiasis cases in non-endemic areas (182). For example, many reports of *S. mansoni* infected cases in immigrants or tourists visiting Romania and neighboring Hungary were reported (183). Also, more than 400 imported cases infected with African schistosomes (including *S. mansoni* and *S. hematobium*) were reported from numerous provinces in China between the years 1979 and 2011, owing to the sharp growth in China aided projects in Africa and labor services exported to Africa. It is estimated that over 1 million Chinese workers are now residing in Africa, increasing the risk of exposure to African schistosomiasis (160). Other possible ways of *S. mansoni* establishment include the introduction of infected snails in the same way by which uninfected ones were introduced and introduction of the parasite through Chinese or European laborers returning from endemic areas or visitors traveling for tourism or trade purposes. Once started it is possible that the life cycle could be maintained through other definitive hosts such as rodents.

### Supporting Transmission of Other Helminth Parasites

In addition to its role as intermediate host for *S. mansoni, Biomphalaria* spp. may also act as intermediate hosts for other parasites of mammals, birds, and fish (Table 2). Moreover, different species of *Biomphalaria* were found naturally infected with cercariae and metacercariae of a wide range of trematodes of unknown life cycle (73, 217). Recently, both *B. straminea* and *B. glabrata* were shown to act as intermediate hosts of *Austrodiplostomum compactum* (Trematoda: Diplostomidae), the causative agent for ocular diplostomiasis in several species of fish in Brazil, a disease which may have potential impacts on native fish species and damage to fish farming. The two species were found infected with strigeid cercariae of the parasite (208). *Biomphalaria* can also act as intermediate hosts for different species of the nematode *Angiostrongylus* that infect domestic animals as well as humans. For example, *Angiostrongylus vasorum*, that parasitizes the right ventricle of the heart and the pulmonary artery of dogs and wild carnivores in different parts of Europe, Canada, South America, and Africa (218, 219), is able to infect *B. glabrata* and *B. tenagophila* under laboratory conditions as evidenced by emergence of cercariae that could infect the vertebrate host (209, 210). The parasite can also cause diseases of the central nervous system in humans (220). *B. glabrata* and *B. tenagophila* act as intermediate hosts for the rat lung worms *Angiostrongylus cantonensis* and *A. costaricensis* the causative agents of eosinophilic meningitis and meningoencephalitis and abdominal angiostrongyliasis, respectively, in humans in many parts of the world (221). Within the snails, the parasite develops into the infective third-stage larvae and humans are infected after ingestion of infected snails (222). Infection with the rodent schistosome *S. rodhaini* were found in natural population of *B. sudanica* in some African countries such as Uganda and Burundi (15). Moreover, natural infections with 11 larvae of different trematode species were found in *B. pfeifferi* and *B. sudanica* from Tanzania (223).

**Table 2:** Trematodes and nematodes parasites using *Biomphalaria* as intermediate host.

**Class**	**Parasite species**	***Biomphalaria* species**	**Type of infection**	**Definitive host**
Trematoda	*Echinostoma caproni*	*B. glabrata*	Experimental (184, 185)	Birds and mammals
		*B. pfeifferi*	Natural (186)	
	*Echinostoma paraensei*	*B. glabrata*	Natural (187, 188)	
		*B. tenagophila*	Experimental (189)	
	*Echinostoma trivolvis*	*B. glabrata*	Experimental (190)	
	*Echinostoma liei*	*B. alexandrina*	Natural (191)	
		*B. glabrata*	Experimental (192)	
	*Echinostoma revolutum*	*B. alexandrina*	Experimental (193)	
		*B. glabrata*	Experimental (194)	
	*Echinostoma rodriguesi*	*B. glabrata*	Experimental (195)	
	*Echinostoma luisreyi*	*B. glabrata*	Experimental (196)	
	*Paryphostomum segregatum*	*B. glabrata*	Experimental (197)	
	*Echinostoma lindoense*	*B. glabrata*	Experimental (197)	
	*Echinostoma macrorchi*	*B. glabrata*	Experimental (198)	
	*Echinostoma barbosai*	*B. straminea*	Experimental (199)	
	*Echinostoma friedi*	*B. straminea*	Experimental (200)	
	*Echinostoma togoensis*	*B. glabrata*	Experimental (201)	
	*Echinoparyphium* spp.	*B. glabrata*	Experimental (202)	
		*B. peregrina*	Natural (203)	
		*B. obstructa*	Natural (204)	
	*Zygocotyle lunata*	*B. peregrina*	Natural (205)	
		*B. tenagophila*	Natural (205)	
		*B. straminea*	Experimental (205)	
		*B. orbignyi*	Experimental (205)	
		*B. oligoza*	Experimental (205)	
	*Ribeiroia* spp.	*B. glabrata*	Natural (206)	Fish and amphibians
		*B. straminea*	Natural (207)	
	*Austrodiplostomum compactum*	*B. straminea*	Natural (208)	
		*B. glabrata*	Natural (208)	
Nematoda	*Angiostrongylus vasorum*	*B. glabrata*	Experimental (209)	Dogs and mammals
		*B. tenagophila*	Experimental (210)	
	*Angiostrongylus costaricensis*	*B. glabrata*	Experimental (211)	
	*Angiostrongylus cantonensis*	*B. glabrata*	Experimental (212)	
		*B. straminea*	Experimental (213)	
		*B. alexandrina*	Natural (214)	
			Experimental (215)	
	*Angiostrongylus siamensis*	*B. glabrata*	Experimental (216)	

## Conclusions

The distribution of the snails will define the potential distribution of schistosomiasis and the likelihood of transmission. Many ecological factors regulate distribution and abundance of *Biomphalaria* spp. Hence, knowledge of the disease transmission is incomplete without available information on the ecology and biology of this intermediate host. There is a continuous need to document the changing and dynamic distribution of *Biomphalaria* due to continued environmental modifications and climatic changes.

Long distance dispersal of *Biomphalaria* is achieved through accidental and/ or deliberate introduction to new geographic areas. Dispersal of snail eggs and uninfected snails will not spread schistosomes, but dispersal of infected snails can spread the disease. Moreover, the new species may enhance disease transmission if it is more susceptible to local parasite than sympatric intermediate hosts. Thus, understanding the geographical origins of introduced snails, their modes of dispersal, and their rates of gene flow might assist efforts to understand the spread and establishment of schistosomiasis in the area of introduction. More studies are needed to identify the introduced species and understand their biology and the combination of morphological and molecular methodologies will be the best approach.

Snail distribution data can be used to develop prediction models for potential schistosomiasis risk areas and risk maps for snail distribution and abundance using species modeling software. For this purpose, geographic coordinates should be collected for utilizing in GIS and remotely sensed data (224). The obtained risk maps can be used to apply the suitable public health strategies, and to guide other fieldwork. Also, the risk map will enable better resource distribution and adequate policies for snail control. The GIS approach is particularly recommended to provide estimates of the potential spread of invasive *Biomphalaria* species using data of occurrences through time that allow a functional estimation of the spread of the invader, which could then be related to rates of ecological changes and vector activity over time.

For better and effective control initiatives for schistosomiasis, periodic malacological surveys are recommended for the detection of *Biomphalaria* in new potential habitats and identification of species and infection status of collected snails. Swift actions should be taken to record any new occurrence of these snail intermediate hosts. Community education is another important intervention to aid control. Populations who are in contact with water for agricultural or other purposes should be able to morphologically identify schistosomiasis intermediate snail hosts and understand their role in disease transmission. A surveillance system could be established to monitor the distribution of snails. This system could be used to assist regional policies in controlling snails via application of molluscicides or other control approaches (225, 226).

## Author Contributions

MRH, DR, and X-NZ: conceived and designed the study, and wrote and edited the review. MRH, DR, and SL: data collection and interpretation. All authors read and approved the final manuscript.

## Conflict of Interest

The authors declare that the research was conducted in the absence of any commercial or financial relationships that could be construed as a potential conflict of interest.
